# Mid-infrared electronic wavelength tuning through intracavity difference-frequency mixing in Cr:ZnSe lasers

**DOI:** 10.1038/s41598-022-20914-0

**Published:** 2022-10-04

**Authors:** Masaki Yumoto, Kentaro Miyata, Yasushi Kawata, Satoshi Wada

**Affiliations:** 1 Photonics Control Technology Team, RIKEN Center for Advanced Photonics, RIKEN, 2-1 Hirosawa, Wako, Saitama 351-0198 Japan; 2 Mid-Infrared Laser Source Laboratory, RIKEN Baton Zone Program, RIKEN, 2-1 Hirosawa, Wako, Saitama 351-0198 Japan

**Keywords:** Solid-state lasers, Mid-infrared photonics

## Abstract

Mid-infrared tunable coherent light sources are used in various laser applications, such as trace gas detection, laser processing, and biomedical diagnostics. This study demonstrates mid-infrared generation in the 8.3–11 µm (i.e., 900–1200 cm^−1^) spectral range by configuring intracavity difference-frequency generation (DFG) using ZnGeP_2_ (ZGP) in an electronically tuned Cr:ZnSe laser. The broad tunability is achieved with the maximum pulse energies exceeding 100 μJ by combining the electronic wavelength tuning with sligh angle adjustments (Δ*θ* < 0.5°) of ZGP under the spectral noncritical phase-matching condition of the nonlinear material. The proposed DFG method is generalized to give access to a significant fraction of the molecular fingerprint region by utilizing selenide compounds (e.g., AgGaSe_2_, CdSe) in addition to ZGP, revealing the remarkable potential of ultrabroadband electronic mid-infrared scanning for numerous spectroscopic applications.

The mid-infrared (IR) region has two distinct regions (3–5 and 8–13 µm), which are known as windows of transparency in the Earth’s atmosphere and are hard to be affected by the influence of water vapor absorption^1^. The molecular fingerprint region in the 6.6–20 µm range (i.e., 500–1500 cm^−1^) contains intense and distinctive spectral patterns of molecules^2^. Thus, the tunable laser sources in the 8–13 µm spectral region, where both the Earth’s atmospheric window and the fingerprinted region overlap, provide considerable advantages for applications in remote sensing and trace gas detection of various gas molecules^3–6^. Among such coherent light sources, the nanosecond pulsed mid-IR lasers with high brightness per wavelength and high wavelength controllability provide high sensitivity and a high signal-to-noise ratio for trace gas detection in cavity-ringdown spectroscopy (CRDS) and photoacoustic spectroscopy (PAS). Combined with microscopic and imaging techniques, the light sources also enable label-free biosensing of cells and tissues^7–9^.

For the realization of tunable nanosecond pulsed laser sources in the 8–13 µm range, nonlinear frequency conversion schemes, including difference-frequency generation (DFG) and optical parametric oscillators (OPOs), provide prominent advantages for continuous and broad mid-IR tunability. Since oxide crystals (e. g., KTiOPO_4_, KTiOAsO_4_, and LiNbO_3_) exhibit strong multiphonon absorption beyond 5 µm^10^, non-oxide semiconductor crystals including AgGaS_2_ (AGS), AgGaSe_2_ (AGSe), CdSe, and ZnGeP_2_ (ZGP) are generally used for the nonlinear processes pumped with 1–2 μm lasers^11^.

For the OPO systems, Miyamoto et al*.* have obtained a mid-IR tunability of 5–10 µm and a sub-mJ pulse energy at 7.7 µm by pumping ZGP with the idler output of a galvano-controlled double-crystal KTP OPO^12^. Boyko et al*.* have achieved a much broader tunability in the 5.8–18 µm range with the maximum pulse energy of 171 µJ at 11.5 µm by configuring an AGSe OPO that is pumped with a Rb:PPKTP OPO output at 1.85 µm^13^. Yang et al*.* recently reported a watt-level mid-IR CdSe OPO operating in the 10–11 µm range by using a Ho:YAG master-oscillator and power amplifier system as a pump source, giving ~ 1 mJ idler pulse energy^14^. For the DFG systems, Haidar et al*.* have demonstrated an idler tunability in the 5–12 µm range with the maximum pulse energy of 25 μJ at ~ 8 μm by mixing the signal and idler outputs of a Nd:YAG laser-pumped KTP OPO in ZGP^15^. Mennerat has established a much higher energy operation (up to 10 mJ) in the 5.8–24 µm range by mixing the signal and idler outputs of a Nd:YAG laser-pumped LiNbO_3_ OPO in CdSe, GaSe, and Tl_3_AsSe_3_^16^. However, all these systems require angle tuning of the nonlinear crystal and/or input-wavelength tuning by rotating a diffraction grating, a prism, etc. to obtain the tunable idler outputs, resulting in a low scanning speed. The temperature tuning of the nonlinear crystal is also possible (e.g., see^17^), but with an even lower scanning speed.

To obtain rapid scanning of the idler wavelength, we have previously introduced an electronically-tuned solid-state laser^18^ into the DFG system^19,20^. The method, so-called electronic wavelength tuning, utilizes the acousto-optic tunable filter (AOTF) to enable mid-IR wavelength tuning without a mechanical rotation of the nonlinear optical crystal and wavelength tuning elements. In addition to the high wavelength-tuning speed, electronic wavelength tuning provides distinct features such as rapid wavelength switching in pulse-to-pulse, random-access switching speed, and high wavelength repeatability compared to other wavelength tuning methods as demonstrated in Refs.^20,21^. Continuous scanning and random-access switching in a 9–12 µm range were realized through the extra-cavity AgGaS_2_-DFG by using an electronically tuned dual-wavelength Ti:Al_2_O_3_ laser as a pump source. The pulse energy was, however, limited to below 0.2 µJ, owing to the low available pump energy as well as the low damage threshold of the nonlinear crystal at the near-IR signal and pump wavelengths^19,20^. Electronic wavelength tuning in a similar spectral range was also demonstrated by Zakel et al*.* by integrating an intracavity CdSe OPO into a Cr:ZnSe laser^22^. Despite the highest pulse energy exceeding 200 µJ at 8.25 µm, the resulting tuning range was very limited, i.e., 8.2–8.8 µm, due to the narrow spectral acceptance of the nonlinear material used. Therefore, the mid-IR tuning range of the previous electronic-tuning methods has been so far limited when operating with high pulse energy.

In this study, we propose intracavity DFG in an electronically tuned Cr:ZnSe (ET-Cr:ZnSe) laser for the realization of rapid wavelength tuning in the 8–13 µm range with the maximum pulse energy exceeding 100 μJ. To eliminate the need for two input laser sources in the general DFG process, the ET-Cr:ZnSe laser field pumped by a Tm:YAG laser is used as the signal beam and subsequently frequency-mixed with the residual pump source in a nonlinear crystal for the mid-IR generation. ZGP was selected as a frequency downconverter for proof of concept because, compared to other commercially available nonlinear materials like AGS, AGSe, and CdSe, it provides the much larger nonlinearity (*d*_*36*_ (9.6 μm) = 75 pm/V) together with the excellent thermal conductivity (35–36 W/mK)^23^ despite the rather poor IR transparency at the idler wavelength above ~ 8.5 μm. Here, we apply the electronic wavelength tuning of the signal beam under the spectral noncritical phase-matching (NCPM) condition realized in ZGP to enable broadband mid-IR spectral tuning by combining the electronic tuning with a slight angle adjustment of the nonlinear material. Thus, this intracavity DFG in the ET-Cr:ZnSe laser provides a simple, cost effective solution for designing practical mid-IR coherent sources having broadband rapid tunability together with power scalability.

## Experimental setup and methods

**Figure 1:**
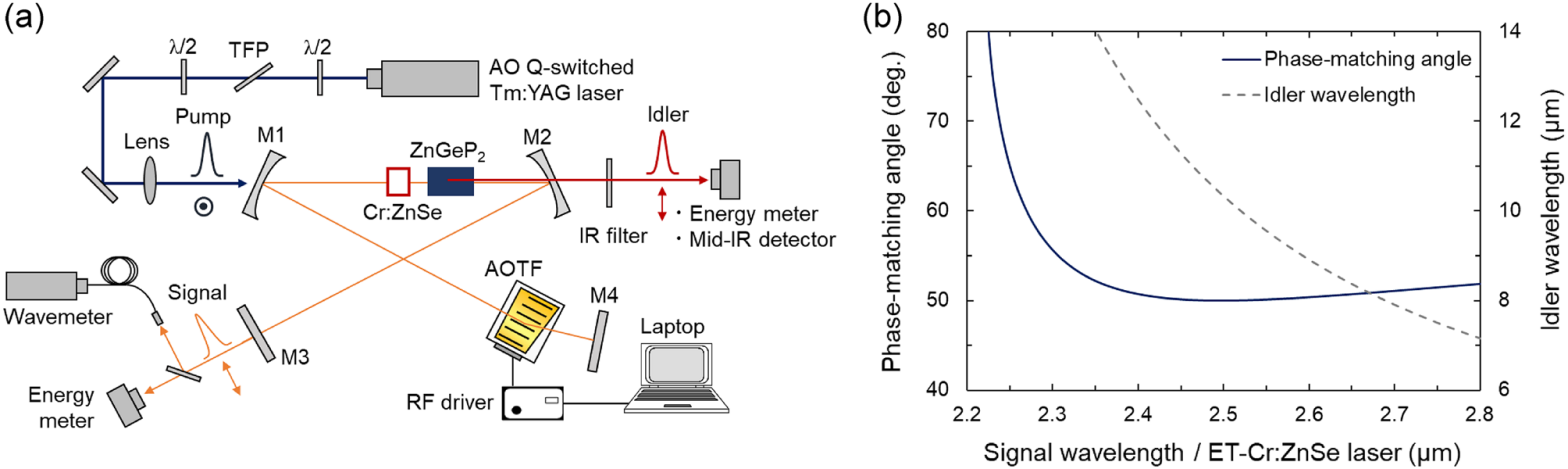
(**a**) Schematic of the experimental setup. TFP, thin-film polarizer: AOTF, acousto-optic tunable filter; M1, folding pump mirror; M2, folding mirror for extracting the idler; M3, output coupler for the ET-Cr:ZnSe laser; and M4, total reflector. (**b**) PM tuning curve for type-1 DFG in ZGP at 20 °C. Pump and signal waves are given by the Tm:YAG laser (2.013 μm) and ET-Cr:ZnSe laser, respectively.

Figure 1a shows a schematic of the experimental setup composed of an acousto-optic (AO) Q-switched Tm:YAG laser, ET-Cr:ZnSe laser, and ZGP-DFG setup. The ET-Cr:ZnSe laser was constructed with a standard X-fold cavity using two CaF_2_ folding mirrors (*R*oc = 500 mm, *R* > 99% at 2.1–3.0 μm, *T* ~ 90% at 2.0 μm), an output coupler (*R* = 95% at 2.1–3.0 μm), a total reflector (*R* > 99% at 2.1–3.0 μm), and the AOTF (Gooch & Housego). As the laser medium, a 5-mm-long antireflection (AR)-coated (for 1.5–2.7 µm) polycrystalline Cr:ZnSe (IPG Photonics, Inc.) having a Cr^2+^ doping concentration of 8.0 × 10^18^ cm^-3^ was placed between two folding mirrors. The pump source was an AO Q-switched Tm:YAG laser (*λ* = 2.013 µm) with a pulse duration of 400 ns at a 10 Hz repetition rate, giving the output pulse energy of 21 mJ, which is varied by an attenuator comprising a half-wave plate and a thin-film polarizer. The output beam of the pump source was loosely focused on the Cr:ZnSe surface with a 1/e^2^ radius of 0.5 mm through M1. The lasing action was confirmed by measuring the pulse energy extracted from the output coupler M3 with an energy meter (PE-10, Ophir) while recording the corresponding central wavelength with a wavemeter (IR-III WS6-200, HighFinesse). Next, a 15-mm-long AR-coated (for 2–3 μm) type-1 ZGP crystal (*θ* = 49°, *φ* = 0°) was placed inside the cavity as a nonlinear frequency downconverter. The pump and signal for the DFG process in ZGP were given by the Tm:YAG laser and the intracavity ET-Cr:ZnSe laser, respectively. Here, the ET-Cr:ZnSe laser enables precise control of the signal wavelength by changing the radio frequency (RF) feeding to the AOTF through the computer program^18^, so that the idler wavelength can be changed synchronously. The pulse energy, beam profile, and pulse width of the idler beam transmitted through M2 (*T*_*avg*_ ~ 60% at 8–11 μm) were measured by using an energy meter (PE-9-ES-C, Ophir), pyroelectric beam profiler (Pyrocam III, Ophir), and mid-IR detector (PVI-4TE-10.6, VIGO system), respectively, after blocking the transmitted pump beam with an IR filter.

Figure 1b shows the type-I (ee-o) PM tuning curve of ZGP for the intracavity-DFG (*λ*_p_ = 2.013 μm) calculated with the Sellmeier equation reported by Kato et al*.*^24^. The idler wavelength is found to be tunable in the 8–12 µm range by varying the signal wavelength in the 2.35–2.50 µm range. At the retracing point (Δ*θ*/Δ*λ*_s_ = 0) of the tuning curve, the inverse group-velocity mismatch between the signal and idler pulses becomes zero (i.e., Δ_si_ = 1∕*ν*_s_ − 1∕*ν*_i_ = 0, where *ν*_s,i_ are group velocities of signal and idler fields)^25^, leading to the broad spectral acceptance for the signal and idler pulses with the fixed pump wavelength. The so-called spectral NCPM condition is generally used for frequency conversion of broadband ultrafast pulsed lasers (see e.g.,^26^) but we apply it to the broadband electronic tuning of the narrow linewidth idler output in this study. In the case of ZGP, the signal and idler wavelengths of the spectral NCPM condition in the present nonlinear process are calculated to be *λ*_s_ = 2.5 µm and *λ*_i_ = 10.5 µm, respectively.

## Experimental results and discussion

### Intracavity-DFG characteristics

**Figure 2:**
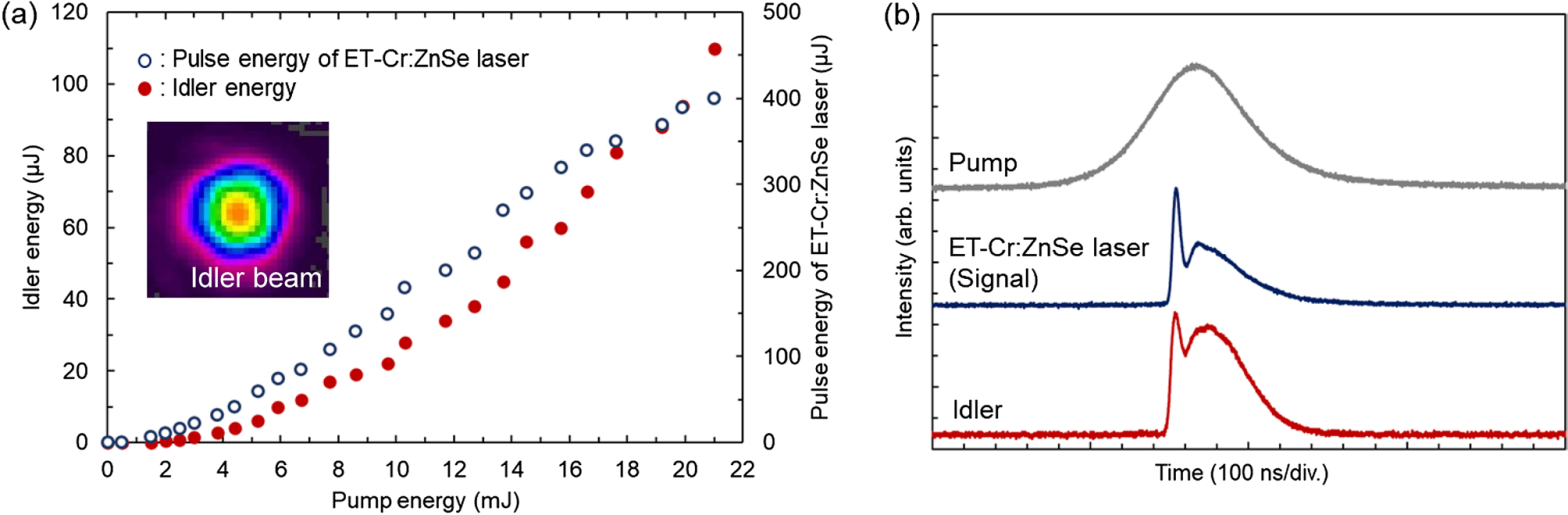
(**a**) Output characteristics of ET-Cr:ZnSe laser (signal beam) and idler beam. The inset shows the idler beam profile at 15 mJ pump energy. (**b**) Temporal profiles of the pump, ET-Cr:ZnSe laser (signal), and idler pulse.

Figure 2a shows the resulting input–output energy dependence at the idler wavelength of 9.0 µm together with the idler beam profile measured at 15 mJ pump energy. The idler pulse energy was detected shortly after the oscillation threshold of the ET-Cr:ZnSe laser (*λ*_s_ = 2.60 µm) and found to nonlinearly increase with the linear increase of the pump and signal pulse energies. The maximum idler energy was 110 µJ at a pump energy of 21 mJ, corresponding to an overall pump-to-idler conversion efficiency of 0.5%. The intracavity signal energy is estimated to be 7.6 mJ by considering the output coupling (*R* = 95% at *λ*_p_) of the measured signal energy of 400 μJ outside the cavity. The intracavity conversion efficiency is then determined to be ~ 2.7% ($={E}_{i}/\sqrt{{E}_{p}\cdot {E}_{s}}$) by using the residual pump energy of 2.3 mJ estimated from the transmitted pump energy of 2.1 mJ that was measured after M2 (*T* = 92% at *λ*_p_). Here, the energy fluences of the pump and signal beams on the ZGP surface were 0.3 and 1.0 J/cm^2^, respectively. No optical damage on the ZGP surface was observed in this condition throughout the experiment.

Note that we have found no clear evidence of the deterioration of the spatial, temporal, and spectral output characteristics for the residual pump source despite the relatively large absorption of the Cr:ZnSe sample used in the excitation process. Hence, the slightly distorted idler beam profile (Fig. 2a) can be attributed to the deep absorption of the ZGP sample at the idler wavelength. Nonetheless, this beam profile is found to be very clean when compared to quantum cascade lasers often used for spectroscopic applications (see, for example, Fig. 2b of Ref.^28^).

The corresponding temporal profile of the idler beam is displayed in Fig. 2b along with those of the input beams. The pump pulse had a Gaussian shape with a duration of ~ 400 ns (FWHM). The temporal profile of the idler pulse duplicates that of the signal pulse modulated by relaxation oscillation of Cr:ZnSe laser^18^, giving the same build-up time of ~ 185 ns. It is possible to suppress the relaxation oscillation to obtain the single peak-pulse operation by increasing the cavity length or decreasing pump pulse duration^27^.

Meanwhile, the spectral widths of the residual pump (i.e., Tm:YAG laser) and signal (i.e., ET-Cr:ZnSe laser) have been measured to be ~ 5 and 1–1.5 cm^−1^, respectively, using a spectrometer (ASP-IR-3.5, AVESTA Ltd.). Thus, the spectral width of the idler beam is expected to be ~ 5 cm^−1^ under the spectral NCPM condition, which agrees well with the estimated values in our gas absorption measurement. Narrowing the pump spectrum can further narrow the idler spectrum.

### Electronic mid-IR wavelength scanning

**Figure 3:**
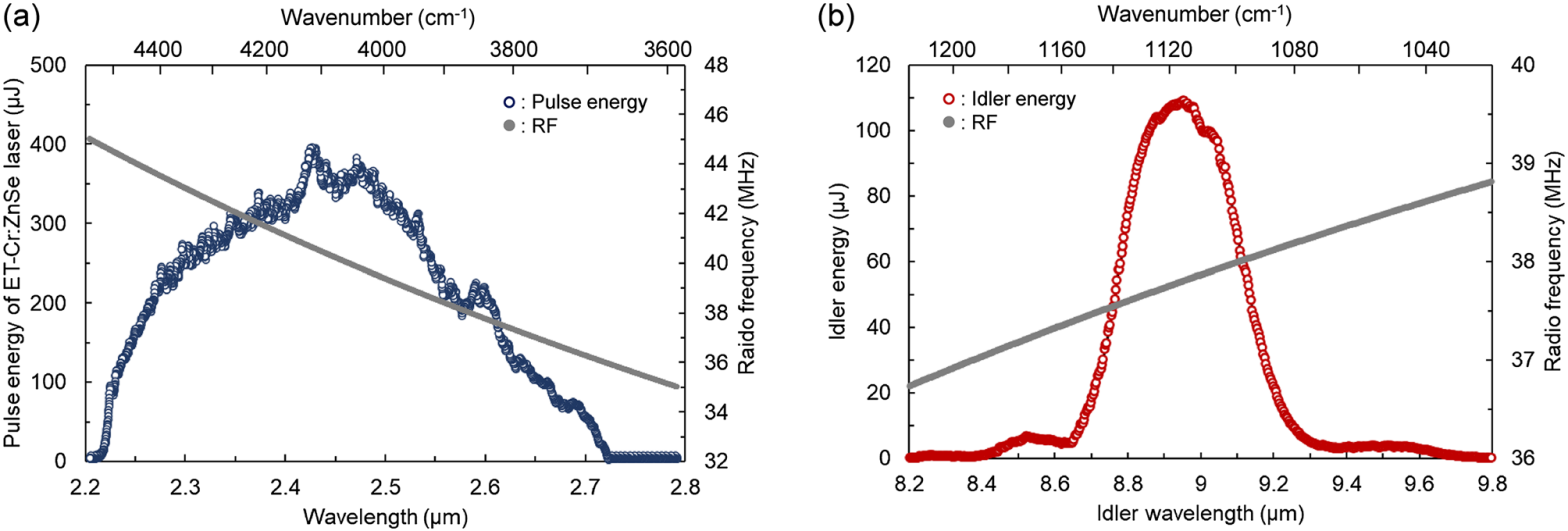
(**a**) Electronic tuning range of the ET-Cr:ZnSe laser and filter tuning curve of the AOTF. (**b**) Electronic tuning range of idler when PM angle was fixed at 50.6°. Gray plots correspond to RFs fed into the AOTF for electronic wavelength tuning.

Figure 3a shows the experimental result of the electronic tuning of the signal wavelength by feeding the RFs to the AOTF with a step size of 0.004 MHz (i.e., Δ*λ*_s_ ~ 0.35 nm) in the 35 and 45 MHz range. The pulse energy at each wavelength was recorded with 1 s averaging at the fixed pump energy of 20 mJ. The signal wavelength was electronically tunable in the 2.22–2.72 µm range, which corresponds to the RF range of 44.7–36.0 MHz. The pulse energy exceeding 250 µJ was obtained around the gain center (2.35–2.5 µm).

Figure 3b shows the resulting tuning range of the idler wavelength. At the fixed PM angle of *θ* = 50.6°, the idler wavelength was tunable in the 8.4–9.7 µm range by electronic tuning the laser wavelength in the 2.54–2.65 µm range (i.e., 37.0–38.7 MHz). The idler pulse energy exceeding 100 µJ was realized in the 8.8–9.0 µm range, which is the highest energy so far reported for mid-IR generation based on the intracavity DFG of the Cr:ZnSe laser. These results show that we achieved high wavelength controllability with high pulse energies in the 8–9 µm range, through intracavity DFG around the spectral NCPM in ZGP combined with the electronic wavelength tuning of the Cr:ZnSe laser.

### Expanding the mid-IR scanning range

**Figure 4:**
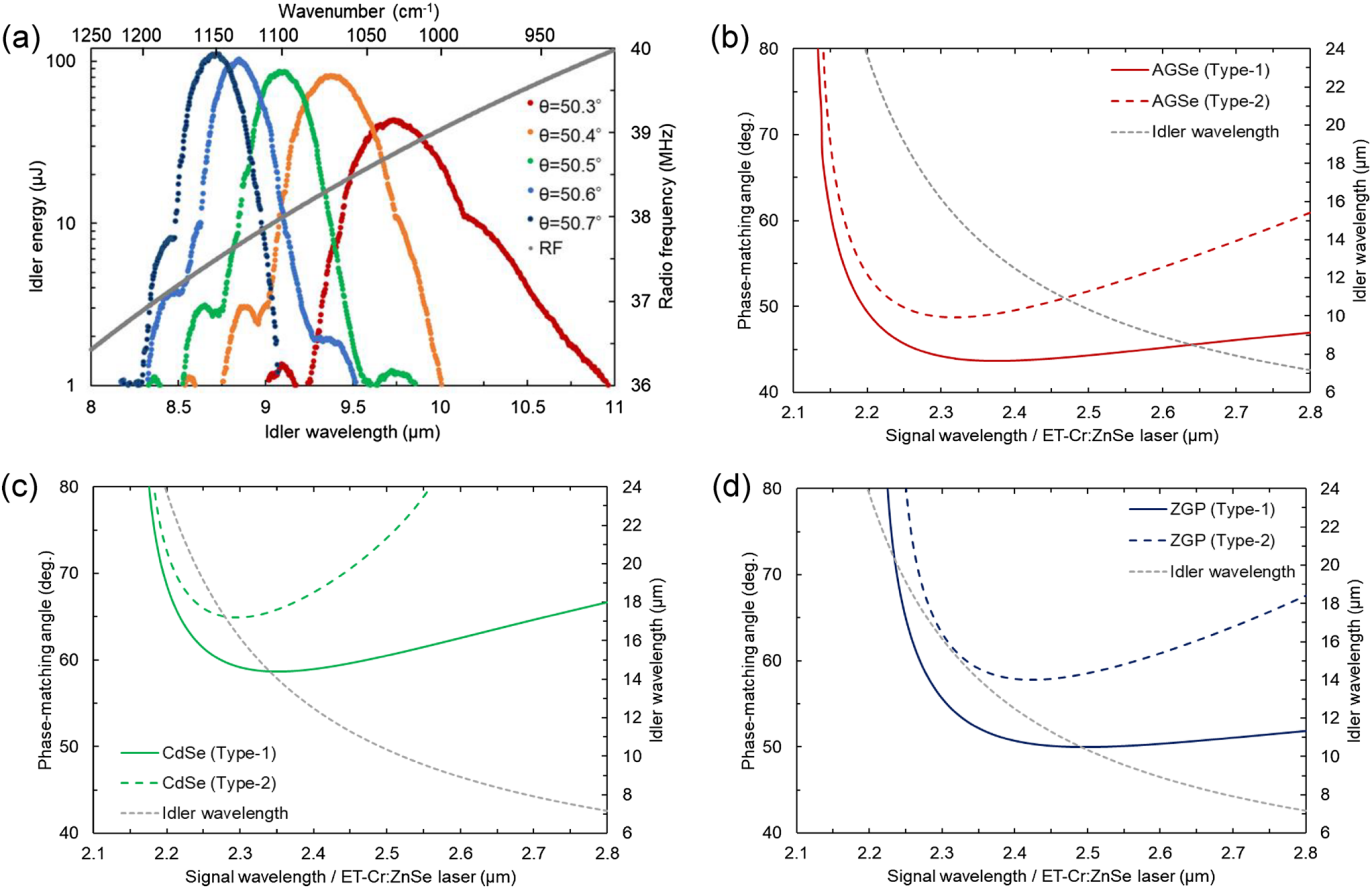
(**a**) Electronic tuning range of idler at PM angle of 50.3°, 50.4°, 50.5°, 50.6°, and 50.7°. Gray plots are RFs fed into the AOTF. (**b**–**d**) PM tuning curves for DFG in AGSe (type-1, 2), CdSe (type-1, 2), and ZGP (type-1, 2) at 20 °C. Pump and signal waves are given by the Tm:YAG laser (2.013 μm) and ET-Cr:ZnSe laser, respectively. The curve was calculated using Sellmeier’s equation of AGSe^29^, CdSe^30^, and ZGP^24^.

It is possible to expand the mid-IR scanning range by combining the electronic wavelength tuning with a slight angle adjustment (Δ*θ* < 0.5°) of the nonlinear crystal around the PM retracing point. Figure 4a shows the electronic idler wavelength tuning by varying the fixed PM angles of ZGP at the pump energy of 20 mJ. The tuning ranges of 8.3–9.2, 8.4–9.5, 8.3–9.9, 8.5–10.1, and 9.0–11.0 µm were realized at the PM angles of 50.3°, 50.4°, 50.5°, 50.6°, and 50.7°, respectively. In the long-wavelength part, the pulse energy was found to decrease owing to the absorption loss of ZGP. However, by selecting only three PM angles of 50.3°, 50.5°, and 50.7°, a broad mid-IR region of 8.3–11 µm can be covered by the electronic tuning of the signal wavelength in the 2.46–2.66 µm range. Here, the signal wavelength was tuned by feeding the RFs to the AOTF with a step size of 0.004 MHz (i.e., Δ*λ*_s_ ~ 0.35 nm) in the 36.9 and 40.0 MHz range.

In the proposed system, the mid-IR scanning range can be further expanded by using different nonlinear materials. For instance, we have calculated the theoretical PM tuning curves of the commercially available nonlinear materials AGSe^29^ and CdSe^30^ for the present DFG process as shown in Fig. 4b. These nonlinear materials are found to have the retracing points of the PM curves within the tuning range of the ET-Cr:ZnSe laser while the effective nonlinearity for the type-1 process of CdSe vanishes because of its structure symmetry. The idler wavelengths of the applicable PM retracing points are *λ*_i_ = 13.0/15.2 µm (*λ*_s_ = 2.38/2.32 µm) for type-1/type-2 AGSe and *λ*_i_ = 16.3 (*λ*_s_ = 2.30 µm) for type-2 CdSe, which are quite longer than *λ*_i_ =  10.4/11.9 µm (*λ*_s_ = 2.50/2.42 µm) for type-1/type-2 ZGP. Thanks to the broad transparency of AGSe (0.71–19 µm^31^) and CdSe (0.75–25 µm^32^), the system therefore enables nonlinear generation of the idler wavelengths well beyond 10 μm without significant absorption losses, where the effectiveness of nonlinear interaction using ZGP is restricted due to the multi-phonon absorptions as already observed in the present experiment (Fig. 4a). Note that the newly discovered selenide compound BaGa_2_GeSe_6_ can be also used as an alternative to AGSe and ZGP to obtain the spectral NCPM condition at *λ*_i_ = 10 and 12 µm for the type-1 and type-2 processes, respectively^33^. Thus, the proposed method is expected to cover the almost entire molecular fingerprint region by using the selenide compounds, in addition to ZGP, in an appropriate manner. Additionally, since the electronic wavelength tuning makes a one-to-one correspondence between RFs, signal wavelengths, and idler wavelengths, the system requires no wavelength monitor to determine the signal and idler wavelengths once the parameter table is obtained. This function is also highly advantageous in tunable mid-IR laser spectroscopy.

## Conclusions

We demonstrated the first intracavity-DFG ET-Cr:ZnSe laser operating in the mid-IR region. The electronic tuning in the 8.3–9.2 µm spectral range was obtained under the spectral NCPM condition of ZGP (*θ* = 50.3°). The corresponding pulse energy has reached 110 μJ at the maximum. The broader tuning range of 8.3–11 µm (i.e., 900–1200 cm^-1^) was also recorded from the same compact setup by combining the electronic tuning with a slight angle adjustment (Δ*θ* < 0.5°) of the nonlinear material. We believe that the proposed method for generating the mid-IR spectra not only simplifies the DFG process by eliminating the need of two input laser sources but provides rapid and broadband wavelength scanning at high energy, which is highly suitable for trace gas detections with CRDS and PAS targeting high-molecular-weight components such as aromatic hydrocarbons (i.e., toluene, and xylene), and refrigerants (i.e., HFC, and CFC) that exhibit broadband mid-IR absorption spectra in the 8–16 µm range.

## Data Availability

The data associated with this research are available from the corresponding author upon reasonable request.
